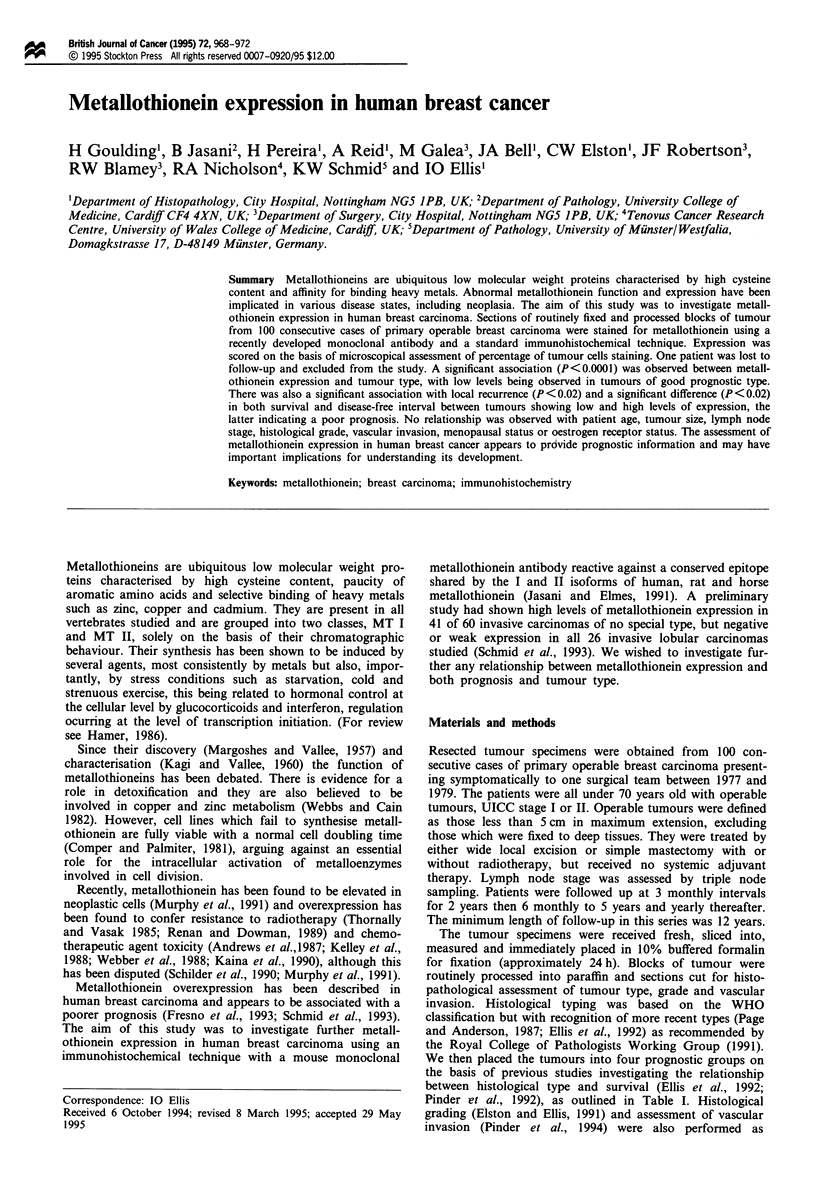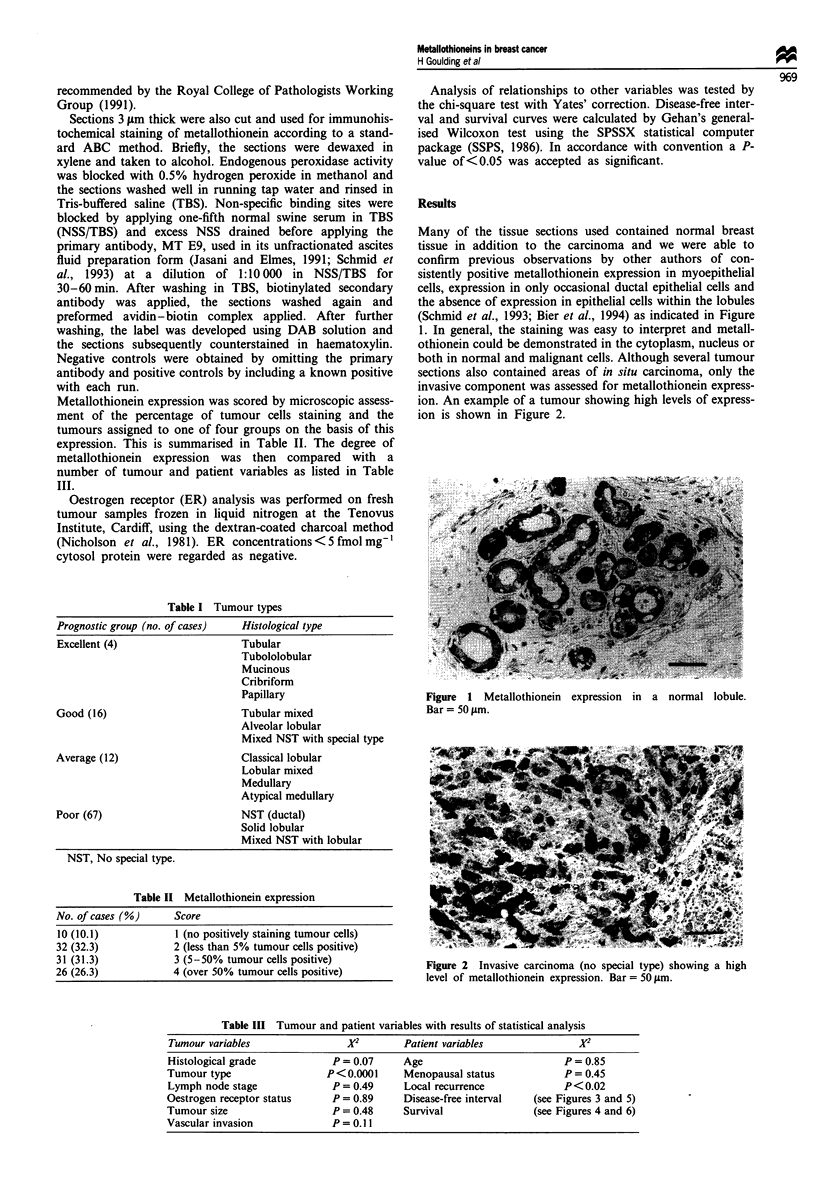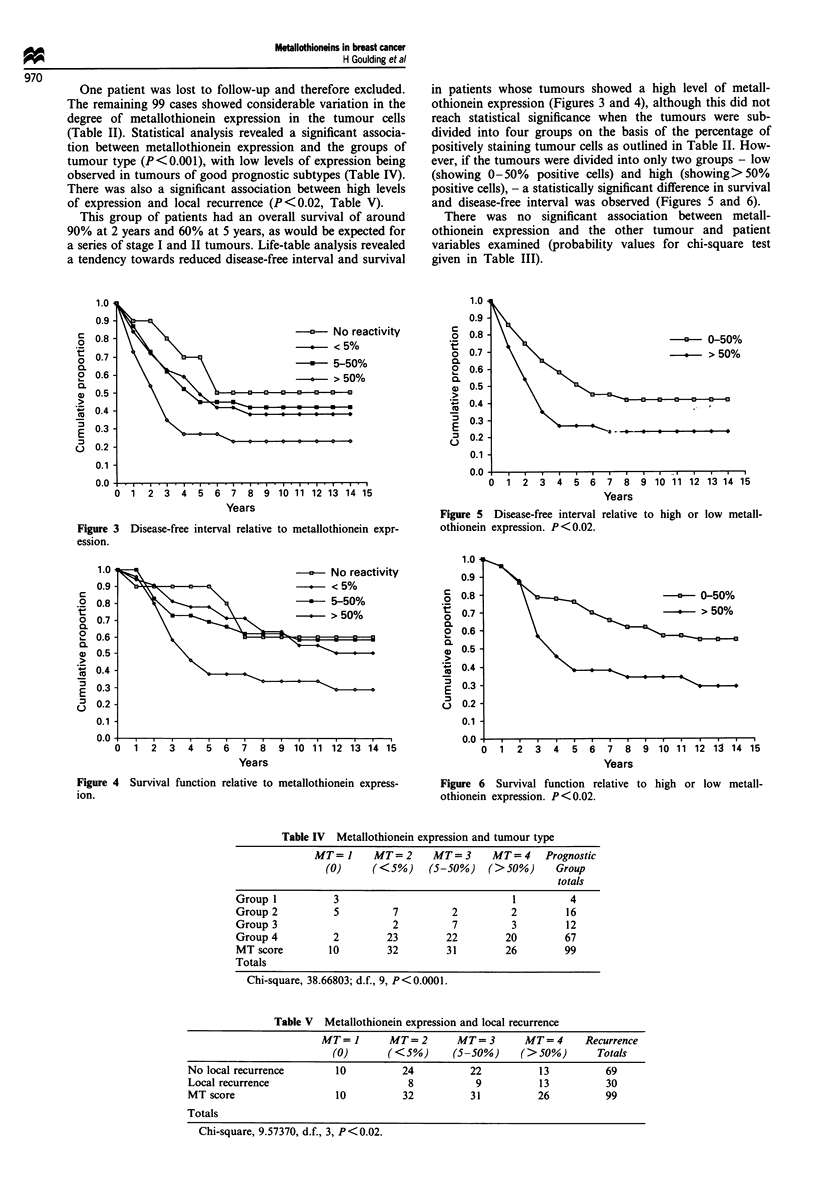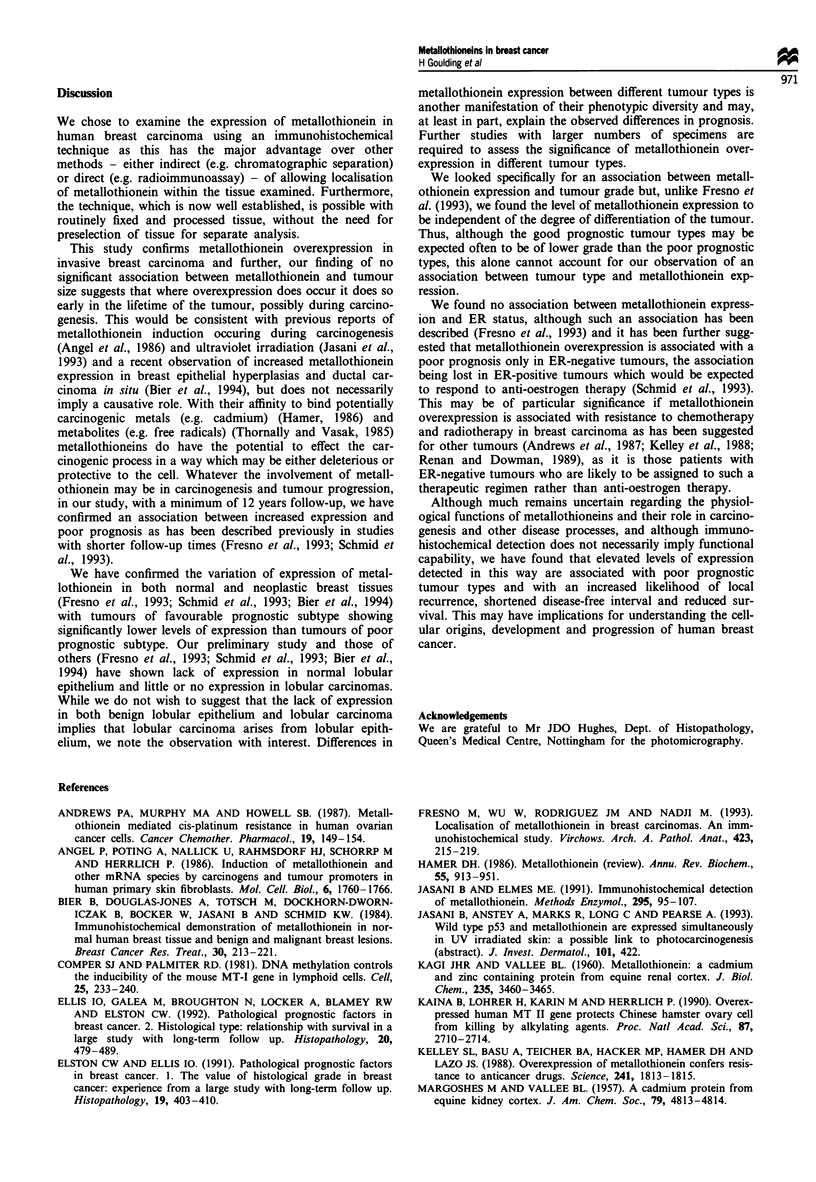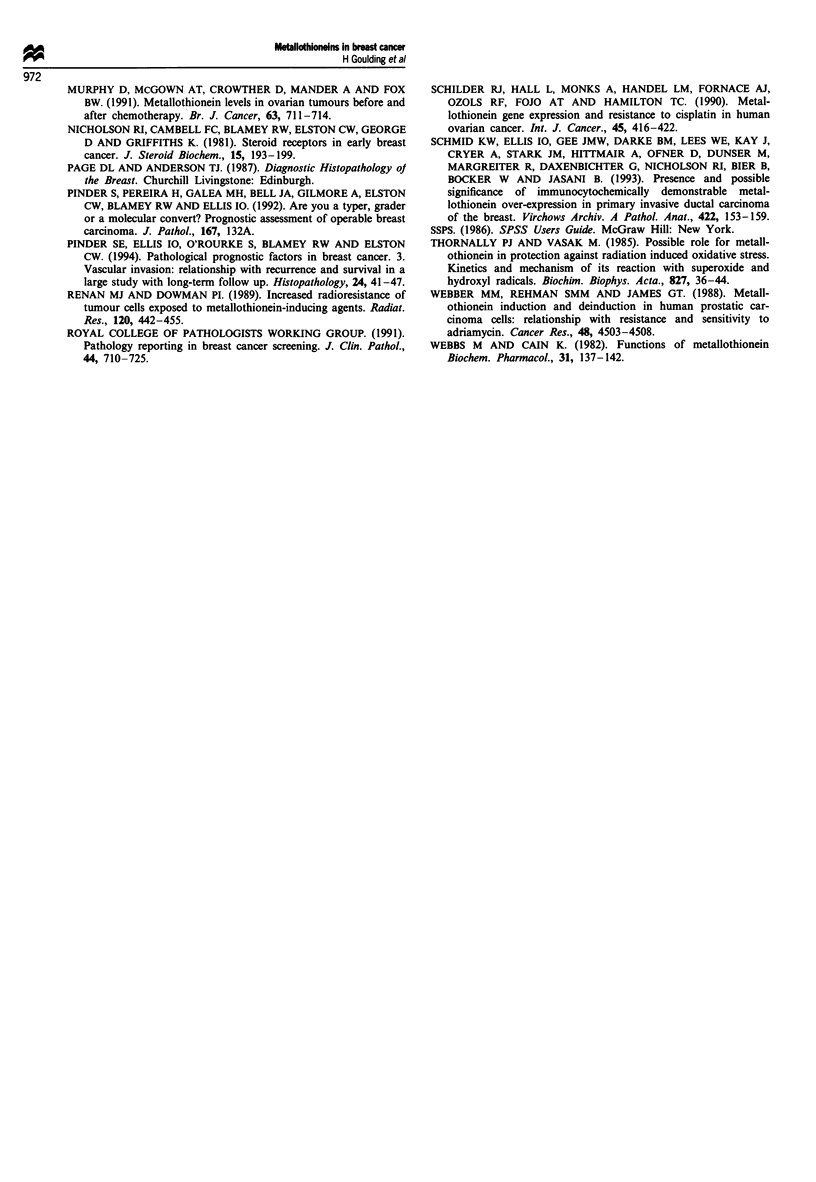# Metallothionein expression in human breast cancer.

**DOI:** 10.1038/bjc.1995.443

**Published:** 1995-10

**Authors:** H. Goulding, B. Jasani, H. Pereira, A. Reid, M. Galea, J. A. Bell, C. W. Elston, J. F. Robertson, R. W. Blamey, R. A. Nicholson

**Affiliations:** Department of Histopathology, City Hospital, Nottingham, UK.

## Abstract

**Images:**


British Journal of Cancer (1995) 72, 968-972
© 1995 Stockton Press All rights reserved 0007-0920/95 $12.00

### Metallothionein expression in human breast cancer

H Goulding[1], B Jasani[2], H Pereira[1], A Reid[1], M Galea[3], JA Bell[1], CW Elston[1], JF Robertson[3], RW Blamey[3], RA Nicholson[4], KW Schmid[5] and IO Ellis[1]

[1]*Department of Histopathology, City Hospital, Nottingham NG5 1PB, UK;* [2]*Department of Pathology, University College of Medicine, Cardiff CF4 4XN, UK;* [3]*Department of Surgery, City Hospital, Nottingham NG5 1PB, UK;* [4]*Tenovus Cancer Research Centre, University of Wales College of Medicine, Cardiff, UK;* [5]*Department of Pathology, University of Münster/Westfalia, Domagkstrasse 17, D-48149 Münster, Germany.*

**Summary** Metallothioneins are ubiquitous low molecular weight proteins characterised by high cysteine content and affinity for binding heavy metals. Abnormal metallothionein function and expression have been implicated in various disease states, including neoplasia. The aim of this study was to investigate metallothionein expression in human breast carcinoma. Sections of routinely fixed and processed blocks of tumour from 100 consecutive cases of primary operable breast carcinoma were stained for metallothionein using a recently developed monoclonal antibody and a standard immunohistochemical technique. Expression was scored on the basis of microscopical assessment of percentage of tumour cells staining. One patient was lost to follow-up and excluded from the study. A significant association ($P < 0.0001$) was observed between metallothionein expression and tumour type, with low levels being observed in tumours of good prognostic type. There was also a significant association with local recurrence ($P < 0.02$) and a significant difference ($P < 0.02$) in both survival and disease-free interval between tumours showing low and high levels of expression, the latter indicating a poor prognosis. No relationship was observed with patient age, tumour size, lymph node stage, histological grade, vascular invasion, menopausal status or oestrogen receptor status. The assessment of metallothionein expression in human breast cancer appears to provide prognostic information and may have important implications for understanding its development.

**Keywords:** metallothionein; breast carcinoma; immunohistochemistry

Metallothioneins are ubiquitous low molecular weight proteins characterised by high cysteine content, paucity of aromatic amino acids and selective binding of heavy metals such as zinc, copper and cadmium. They are present in all vertebrates studied and are grouped into two classes, MT I and MT II, solely on the basis of their chromatographic behaviour. Their synthesis has been shown to be induced by several agents, most consistently by metals but also, importantly, by stress conditions such as starvation, cold and strenuous exercise, this being related to hormonal control at the cellular level by glucocorticoids and interferon, regulation ocurring at the level of transcription initiation. (For review see Hamer, 1986).

Since their discovery (Margoshes and Vallee, 1957) and characterisation (Kagi and Vallee, 1960) the function of metallothioneins has been debated. There is evidence for a role in detoxification and they are also believed to be involved in copper and zinc metabolism (Webbs and Cain 1982). However, cell lines which fail to synthesise metallothionein are fully viable with a normal cell doubling time (Comper and Palmiter, 1981), arguing against an essential role for the intracellular activation of metalloenzymes involved in cell division.

Recently, metallothionein has been found to be elevated in neoplastic cells (Murphy *et al.*, 1991) and overexpression has been found to confer resistance to radiotherapy (Thornally and Vasak 1985; Renan and Dowman, 1989) and chemotherapeutic agent toxicity (Andrews *et al.*,1987; Kelley *et al.*, 1988; Webber *et al.*, 1988; Kaina *et al.*, 1990), although this has been disputed (Schilder *et al.*, 1990; Murphy *et al.*, 1991).

Metallothionein overexpression has been described in human breast carcinoma and appears to be associated with a poorer prognosis (Fresno *et al.*, 1993; Schmid *et al.*, 1993). The aim of this study was to investigate further metallothionein expression in human breast carcinoma using an immunohistochemical technique with a mouse monoclonal metallothionein antibody reactive against a conserved epitope shared by the I and II isoforms of human, rat and horse metallothionein (Jasani and Elmes, 1991). A preliminary study had shown high levels of metallothionein expression in 41 of 60 invasive carcinomas of no special type, but negative or weak expression in all 26 invasive lobular carcinomas studied (Schmid *et al.*, 1993). We wished to investigate further any relationship between metallothionein expression and both prognosis and tumour type.

Correspondence: IO Ellis
Received 6 October 1994; revised 8 March 1995; accepted 29 May 1995

### Materials and methods

Resected tumour specimens were obtained from 100 consecutive cases of primary operable breast carcinoma presenting symptomatically to one surgical team between 1977 and 1979. The patients were all under 70 years old with operable tumours, UICC stage I or II. Operable tumours were defined as those less than 5 cm in maximum extension, excluding those which were fixed to deep tissues. They were treated by either wide local excision or simple mastectomy with or without radiotherapy, but received no systemic adjuvant therapy. Lymph node stage was assessed by triple node sampling. Patients were followed up at 3 monthly intervals for 2 years then 6 monthly to 5 years and yearly thereafter. The minimum length of follow-up in this series was 12 years.

The tumour specimens were received fresh, sliced into, measured and immediately placed in 10% buffered formalin for fixation (approximately 24 h). Blocks of tumour were routinely processed into paraffin and sections cut for histopathological assessment of tumour type, grade and vascular invasion. Histological typing was based on the WHO classification but with recognition of more recent types (Page and Anderson, 1987; Ellis *et al.*, 1992) as recommended by the Royal College of Pathologists Working Group (1991). We then placed the tumours into four prognostic groups on the basis of previous studies investigating the relationship between histological type and survival (Ellis *et al.*, 1992; Pinder *et al.*, 1992), as outlined in Table I. Histological grading (Elston and Ellis, 1991) and assessment of vascular invasion (Pinder *et al.*, 1994) were also performed as recommended by the Royal College of Pathologists Working Group (1991).

Sections 3 μm thick were also cut and used for immunohistochemical staining of metallothionein according to a standard ABC method. Briefly, the sections were dewaxed in xylene and taken to alcohol. Endogenous peroxidase activity was blocked with 0.5% hydrogen peroxide in methanol and the sections washed well in running tap water and rinsed in Tris-buffered saline (TBS). Non-specific binding sites were blocked by applying one-fifth normal swine serum in TBS (NSS/TBS) and excess NSS drained before applying the primary antibody, MT E9, used in its unfractionated ascites fluid preparation form (Jasani and Elmes, 1991; Schmid *et al*., 1993) at a dilution of 1:10 000 in NSS/TBS for 30–60 min. After washing in TBS, biotinylated secondary antibody was applied, the sections washed again and preformed avidin–biotin complex applied. After further washing, the label was developed using DAB solution and the sections subsequently counterstained in haematoxylin. Negative controls were obtained by omitting the primary antibody and positive controls by including a known positive with each run.

Metallothionein expression was scored by microscopic assessment of the percentage of tumour cells staining and the tumours assigned to one of four groups on the basis of this expression. This is summarised in Table II. The degree of metallothionein expression was then compared with a number of tumour and patient variables as listed in Table III.

Oestrogen receptor (ER) analysis was performed on fresh tumour samples frozen in liquid nitrogen at the Tenovus Institute, Cardiff, using the dextran-coated charcoal method (Nicholson *et al*., 1981). ER concentrations $< 5\ \text{fmol mg}^{-1}$ cytosol protein were regarded as negative.

**Table I** Tumour types

| *Prognostic group (no. of cases)* | *Histological type* |
|---|---|
| Excellent (4) | Tubular |
| | Tubololobular |
| | Mucinous |
| | Cribriform |
| | Papillary |
| Good (16) | Tubular mixed |
| | Alveolar lobular |
| | Mixed NST with special type |
| Average (12) | Classical lobular |
| | Lobular mixed |
| | Medullary |
| | Atypical medullary |
| Poor (67) | NST (ductal) |
| | Solid lobular |
| | Mixed NST with lobular |

NST, No special type.

**Table II** Metallothionein expression

| *No. of cases (%)* | *Score* |
|---|---|
| 10 (10.1) | 1 (no positively staining tumour cells) |
| 32 (32.3) | 2 (less than 5% tumour cells positive) |
| 31 (31.3) | 3 (5–50% tumour cells positive) |
| 26 (26.3) | 4 (over 50% tumour cells positive) |

Analysis of relationships to other variables was tested by the chi-square test with Yates' correction. Disease-free interval and survival curves were calculated by Gehan's generalised Wilcoxon test using the SPSSX statistical computer package (SSPS, 1986). In accordance with convention a *P*-value of $< 0.05$ was accepted as significant.

### Results

Many of the tissue sections used contained normal breast tissue in addition to the carcinoma and we were able to confirm previous observations by other authors of consistently positive metallothionein expression in myoepithelial cells, expression in only occasional ductal epithelial cells and the absence of expression in epithelial cells within the lobules (Schmid *et al*., 1993; Bier *et al*., 1994) as indicated in Figure 1. In general, the staining was easy to interpret and metallothionein could be demonstrated in the cytoplasm, nucleus or both in normal and malignant cells. Although several tumour sections also contained areas of *in situ* carcinoma, only the invasive component was assessed for metallothionein expression. An example of a tumour showing high levels of expression is shown in Figure 2.

**Figure 1** Metallothionein expression in a normal lobule. Bar = 50 μm.

**Figure 2** Invasive carcinoma (no special type) showing a high level of metallothionein expression. Bar = 50 μm.

**Table III** Tumour and patient variables with results of statistical analysis

| *Tumour variables* | *$X^2$* | *Patient variables* | *$X^2$* |
|---|---|---|---|
| Histological grade | $P = 0.07$ | Age | $P = 0.85$ |
| Tumour type | $P < 0.0001$ | Menopausal status | $P = 0.45$ |
| Lymph node stage | $P = 0.49$ | Local recurrence | $P < 0.02$ |
| Oestrogen receptor status | $P = 0.89$ | Disease-free interval | (see Figures 3 and 5) |
| Tumour size | $P = 0.48$ | Survival | (see Figures 4 and 6) |
| Vascular invasion | $P = 0.11$ | | |

One patient was lost to follow-up and therefore excluded. The remaining 99 cases showed considerable variation in the degree of metallothionein expression in the tumour cells (Table II). Statistical analysis revealed a significant association between metallothionein expression and the groups of tumour type ($P<0.001$), with low levels of expression being observed in tumours of good prognostic subtypes (Table IV). There was also a significant association between high levels of expression and local recurrence ($P<0.02$, Table V).

This group of patients had an overall survival of around 90% at 2 years and 60% at 5 years, as would be expected for a series of stage I and II tumours. Life-table analysis revealed a tendency towards reduced disease-free interval and survival in patients whose tumours showed a high level of metallothionein expression (Figures 3 and 4), although this did not reach statistical significance when the tumours were subdivided into four groups on the basis of the percentage of positively staining tumour cells as outlined in Table II. However, if the tumours were divided into only two groups – low (showing 0–50% positive cells) and high (showing>50% positive cells), – a statistically significant difference in survival and disease-free interval was observed (Figures 5 and 6).

There was no significant association between metallothionein expression and the other tumour and patient variables examined (probability values for chi-square test given in Table III).

**Figure 3** Disease-free interval relative to metallothionein expression.

**Figure 4** Survival function relative to metallothionein expression.

**Figure 5** Disease-free interval relative to high or low metallothionein expression. $P<0.02$.

**Figure 6** Survival function relative to high or low metallothionein expression. $P<0.02$.

**Table IV** Metallothionein expression and tumour type

| | *MT=1 (0)* | *MT=2 (<5%)* | *MT=3 (5–50%)* | *MT=4 (>50%)* | *Prognostic Group totals* |
|---|---|---|---|---|---|
| Group 1 | 3 | | | 1 | 4 |
| Group 2 | 5 | 7 | 2 | 2 | 16 |
| Group 3 | | 2 | 7 | 3 | 12 |
| Group 4 | 2 | 23 | 22 | 20 | 67 |
| MT score Totals | 10 | 32 | 31 | 26 | 99 |

Chi-square, 38.66803; d.f., 9, $P<0.0001$.

**Table V** Metallothionein expression and local recurrence

| | *MT=1 (0)* | *MT=2 (<5%)* | *MT=3 (5–50%)* | *MT=4 (>50%)* | *Recurrence Totals* |
|---|---|---|---|---|---|
| No local recurrence | 10 | 24 | 22 | 13 | 69 |
| Local recurrence | | 8 | 9 | 13 | 30 |
| MT score | 10 | 32 | 31 | 26 | 99 |
| Totals | | | | | |

Chi-square, 9.57370, d.f., 3, $P<0.02$.

### Discussion

We chose to examine the expression of metallothionein in human breast carcinoma using an immunohistochemical technique as this has the major advantage over other methods – either indirect (e.g. chromatographic separation) or direct (e.g. radioimmunoassay) – of allowing localisation of metallothionein within the tissue examined. Furthermore, the technique, which is now well established, is possible with routinely fixed and processed tissue, without the need for preselection of tissue for separate analysis.

This study confirms metallothionein overexpression in invasive breast carcinoma and further, our finding of no significant association between metallothionein and tumour size suggests that where overexpression does occur it does so early in the lifetime of the tumour, possibly during carcinogenesis. This would be consistent with previous reports of metallothionein induction occuring during carcinogenesis (Angel *et al.*, 1986) and ultraviolet irradiation (Jasani *et al.*, 1993) and a recent observation of increased metallothionein expression in breast epithelial hyperplasias and ductal carcinoma *in situ* (Bier *et al.*, 1994), but does not necessarily imply a causative role. With their affinity to bind potentially carcinogenic metals (e.g. cadmium) (Hamer, 1986) and metabolites (e.g. free radicals) (Thornally and Vasak, 1985) metallothioneins do have the potential to effect the carcinogenic process in a way which may be either deleterious or protective to the cell. Whatever the involvement of metallothionein may be in carcinogenesis and tumour progression, in our study, with a minimum of 12 years follow-up, we have confirmed an association between increased expression and poor prognosis as has been described previously in studies with shorter follow-up times (Fresno *et al.*, 1993; Schmid *et al.*, 1993).

We have confirmed the variation of expression of metallothionein in both normal and neoplastic breast tissues (Fresno *et al.*, 1993; Schmid *et al.*, 1993; Bier *et al.*, 1994) with tumours of favourable prognostic subtype showing significantly lower levels of expression than tumours of poor prognostic subtype. Our preliminary study and those of others (Fresno *et al.*, 1993; Schmid *et al.*, 1993; Bier *et al.*, 1994) have shown lack of expression in normal lobular epithelium and little or no expression in lobular carcinomas. While we do not wish to suggest that the lack of expression in both benign lobular epithelium and lobular carcinoma implies that lobular carcinoma arises from lobular epithelium, we note the observation with interest. Differences in metallothionein expression between different tumour types is another manifestation of their phenotypic diversity and may, at least in part, explain the observed differences in prognosis. Further studies with larger numbers of specimens are required to assess the significance of metallothionein overexpression in different tumour types.

We looked specifically for an association between metallothionein expression and tumour grade but, unlike Fresno *et al.* (1993), we found the level of metallothionein expression to be independent of the degree of differentiation of the tumour. Thus, although the good prognostic tumour types may be expected often to be of lower grade than the poor prognostic types, this alone cannot account for our observation of an association between tumour type and metallothionein expression.

We found no association between metallothionein expression and ER status, although such an association has been described (Fresno *et al.*, 1993) and it has been further suggested that metallothionein overexpression is associated with a poor prognosis only in ER-negative tumours, the association being lost in ER-positive tumours which would be expected to respond to anti-oestrogen therapy (Schmid *et al.*, 1993). This may be of particular significance if metallothionein overexpression is associated with resistance to chemotherapy and radiotherapy in breast carcinoma as has been suggested for other tumours (Andrews *et al.*, 1987; Kelley *et al.*, 1988; Renan and Dowman, 1989), as it is those patients with ER-negative tumours who are likely to be assigned to such a therapeutic regimen rather than anti-oestrogen therapy.

Although much remains uncertain regarding the physiological functions of metallothioneins and their role in carcinogenesis and other disease processes, and although immunohistochemical detection does not necessarily imply functional capability, we have found that elevated levels of expression detected in this way are associated with poor prognostic tumour types and with an increased likelihood of local recurrence, shortened disease-free interval and reduced survival. This may have implications for understanding the cellular origins, development and progression of human breast cancer.

**Acknowledgements**
We are grateful to Mr JDO Hughes, Dept. of Histopathology, Queen's Medical Centre, Nottingham for the photomicrography.

### References

ANDREWS PA, MURPHY MA AND HOWELL SB. (1987). Metallothionein mediated cis-platinum resistance in human ovarian cancer cells. *Cancer Chemother. Pharmacol.*, **19**, 149–154.

ANGEL P, POTING A, NALLICK U, RAHMSDORF HJ, SCHORRP M AND HERRLICH P. (1986). Induction of metallothionein and other mRNA species by carcinogens and tumour promoters in human primary skin fibroblasts. *Mol. Cell. Biol.*, **6**, 1760–1766.

BIER B, DOUGLAS-JONES A, TOTSCH M, DOCKHORN-DWORNICZAK B, BOCKER W, JASANI B AND SCHMID KW. (1984). Immunohistochemical demonstration of metallothionein in normal human breast tissue and benign and malignant breast lesions. *Breast Cancer Res. Treat.*, **30**, 213–221.

COMPER SJ AND PALMITER RD. (1981). DNA methylation controls the inducibility of the mouse MT-I gene in lymphoid cells. *Cell*, **25**, 233–240.

ELLIS IO, GALEA M, BROUGHTON N, LOCKER A, BLAMEY RW AND ELSTON CW. (1992). Pathological prognostic factors in breast cancer. 2. Histological type: relationship with survival in a large study with long-term follow up. *Histopathology*, **20**, 479–489.

ELSTON CW AND ELLIS IO. (1991). Pathological prognostic factors in breast cancer. 1. The value of histological grade in breast cancer: experience from a large study with long-term follow up. *Histopathology*, **19**, 403–410.

FRESNO M, WU W, RODRIGUEZ JM AND NADJI M. (1993). Localisation of metallothionein in breast carcinomas. An immunohistochemical study. *Virchows. Arch. A. Pathol. Anat.*, **423**, 215–219.

HAMER DH. (1986). Metallothionein (review). *Annu. Rev. Biochem.*, **55**, 913–951.

JASANI B AND ELMES ME. (1991). Immunohistochemical detection of metallothionein. *Methods Enzymol.*, **295**, 95–107.

JASANI B, ANSTEY A, MARKS R, LONG C AND PEARSE A. (1993). Wild type p53 and metallothionein are expressed simultaneously in UV irradiated skin: a possible link to photocarcinogenesis (abstract). *J. Invest. Dermatol.*, **101**, 422.

KAGI JHR AND VALLEE BL. (1960). Metallothionein: a cadmium and zinc containing protein from equine renal cortex. *J. Biol. Chem.*, **235**, 3460–3465.

KAINA B, LOHRER H, KARIN M AND HERRLICH P. (1990). Overexpressed human MT II gene protects Chinese hamster ovary cell from killing by alkylating agents. *Proc. Natl Acad. Sci.*, **87**, 2710–2714.

KELLEY SL, BASU A, TEICHER BA, HACKER MP, HAMER DH AND LAZO JS. (1988). Overexpression of metallothionein confers resistance to anticancer drugs. *Science*, **241**, 1813–1815.

MARGOSHES M AND VALLEE BL. (1957). A cadmium protein from equine kidney cortex. *J. Am. Chem. Soc.*, **79**, 4813–4814.

MURPHY D, MCGOWN AT, CROWTHER D, MANDER A AND FOX BW. (1991). Metallothionein levels in ovarian tumours before and after chemotherapy. *Br. J. Cancer*, **63**, 711–714.

NICHOLSON RI, CAMBELL FC, BLAMEY RW, ELSTON CW, GEORGE D AND GRIFFITHS K. (1981). Steroid receptors in early breast cancer. *J. Steroid Biochem.*, **15**, 193–199.

PAGE DL AND ANDERSON TJ. (1987). *Diagnostic Histopathology of the Breast*. Churchill Livingstone: Edinburgh.

PINDER S, PEREIRA H, GALEA MH, BELL JA, GILMORE A, ELSTON CW, BLAMEY RW AND ELLIS IO. (1992). Are you a typer, grader or a molecular convert? Prognostic assessment of operable breast carcinoma. *J. Pathol.*, **167**, 132A.

PINDER SE, ELLIS IO, O'ROURKE S, BLAMEY RW AND ELSTON CW. (1994). Pathological prognostic factors in breast cancer. 3. Vascular invasion: relationship with recurrence and survival in a large study with long-term follow up. *Histopathology*, **24**, 41–47.

RENAN MJ AND DOWMAN PI. (1989). Increased radioresistance of tumour cells exposed to metallothionein-inducing agents. *Radiat. Res.*, **120**, 442–455.

ROYAL COLLEGE OF PATHOLOGISTS WORKING GROUP. (1991). Pathology reporting in breast cancer screening. *J. Clin. Pathol.*, **44**, 710–725.

SCHILDER RJ, HALL L, MONKS A, HANDEL LM, FORNACE AJ, OZOLS RF, FOJO AT AND HAMILTON TC. (1990). Metallothionein gene expression and resistance to cisplatin in human ovarian cancer. *Int. J. Cancer.*, **45**, 416–422.

SCHMID KW, ELLIS IO, GEE JMW, DARKE BM, LEES WE, KAY J, CRYER A, STARK JM, HITTMAIR A, OFNER D, DUNSER M, MARGREITER R, DAXENBICHTER G, NICHOLSON RI, BIER B, BOCKER W AND JASANI B. (1993). Presence and possible significance of immunocytochemically demonstrable metallothionein over-expression in primary invasive ductal carcinoma of the breast. *Virchows Archiv. A Pathol. Anat.*, **422**, 153–159.

SSPS. (1986). *SPSS Users Guide*. McGraw Hill: New York.

THORNALLY PJ AND VASAK M. (1985). Possible role for metallothionein in protection against radiation induced oxidative stress. Kinetics and mechanism of its reaction with superoxide and hydroxyl radicals. *Biochim. Biophys. Acta.*, **827**, 36–44.

WEBBER MM, REHMAN SMM AND JAMES GT. (1988). Metallothionein induction and deinduction in human prostatic carcinoma cells: relationship with resistance and sensitivity to adriamycin. *Cancer Res.*, **48**, 4503–4508.

WEBBS M AND CAIN K. (1982). Functions of metallothionein *Biochem. Pharmacol.*, **31**, 137–142.